# A Novel Orthogonal Waveform Separation Scheme for Airborne MIMO-SAR Systems

**DOI:** 10.3390/s18103580

**Published:** 2018-10-22

**Authors:** Jie Wang, Ke-Hong Zhu, Li-Na Wang, Xing-Dong Liang, Long-Yong Chen

**Affiliations:** 1 School of Electronic & Information Engineering, Nanjing University of Information Science & Technology, Nanjing 210044, China; 001283@nuist.edu.cn; 2 Science and Technology on Microwave Imaging Laboratory, Institute of Electronics, Chinese Academy of Sciences, Beijing 100190, China; tyler523@163.com (K.-H.Z.); lychen@mail.ie.ac.cn (L.-Y.C.)

**Keywords:** airborne multi-input multi-output (MIMO) radar, synthetic aperture radar (SAR) systems, orthogonal waveform separation, echo-compression

## Abstract

In recent years, multi-input multi-output (MIMO) synthetic aperture radar (SAR) systems, which can promote the performance of 3D imaging, high-resolution wide-swath remote sensing, and multi-baseline interferometry, have received considerable attention. Several papers on MIMO-SAR have been published, but the research of such systems is seriously limited. This is mainly because the superposed echoes of the multiple transmitted orthogonal waveforms cannot be separated perfectly. The imperfect separation will introduce ambiguous energy and degrade SAR images dramatically. In this paper, a novel orthogonal waveform separation scheme based on echo-compression is proposed for airborne MIMO-SAR systems. Specifically, apart from the simultaneous transmissions, the transmitters are required to radiate several times alone in a synthetic aperture to sense their private inner-aperture channels. Since the channel responses at the neighboring azimuth positions are relevant, the energy of the solely radiated orthogonal waveforms in the superposed echoes will be concentrated. To this end, the echoes of the multiple transmitted orthogonal waveforms can be separated by cancelling the peaks. In addition, the cleaned echoes, along with original superposed one, can be used to reconstruct the unambiguous echoes. The proposed scheme is validated by simulations.

## 1. Introduction

Synthetic aperture radar (SAR) systems have been extensively contributing to diverse scientific applications and to remote sensing missions [1]. Expanding the performance of SAR systems, capabilities of wide coverage, fine geometric resolution, and multimodal operation become more crucial for future SAR missions [2]. However, performance of conventional multichannel radar systems cannot meet the forthcoming surveillance or reconnaissance requirements due to its restricted degrees of freedom in transmission, let alone the single-antenna systems. Consequently, multi-input multi-output (MIMO) SAR concepts have emerged as a new paradigm for future SAR systems [3,4]. This radar can acquire more degrees of freedom (waveform diversities, massive phase centers et al.) by simultaneously transmitting mutually orthogonal waveforms in the same frequency band [5]. To this end, it becomes possible to break though the systematic limit of traditional SAR to realize high-resolution wide-swath imaging, multimodal operation, and 3D imaging etc. Although MIMO-SAR has received considerable attention in recent years [6,7,8,9,10], the research of such systems is still limited by imperfect orthogonal waveforms [11]. Some authors have suggested the up and down chirps [6] (or more sophisticated waveforms). For these waveforms, the energy of the multiple orthogonal waveforms is spread but not removed in the time-frequency plane [12]. This ambiguous energy, which would be accumulated from numerous spatially distributed targets in the scene, increases the background noise level in both range and azimuth directions. Due to the raised background noise level, the imperfect waveform separation introduced ambiguous energy degrades MIMO-SAR images dramatically. 

To avoid the ambiguous energy, the multidimensional waveforms such as the short-term shift-orthogonal (STSO) waveforms, the orthogonal frequency division multiplexing (OFDM) chirp waveforms and the inter-pulse phase modulation (IPPM) with Range-Doppler decouple filtering schemes have emerged as potential solutions [13,14,15,16]. However, both STSO waveforms and OFDM chirp waveforms employ digital beam forming (DBF) technique in elevation direction. This requirement would increase the system complexity and cost substantially. For example, in [2], the designed system requires 42 subarrays in the elevation direction to perform the spatial filtering. As for the IPPM with Range-Doppler decouple filtering scheme, which modulates two transmitted signals into distinct Doppler center frequencies, it would double the pulse repeat frequency (PRF) and reduce MIMO-SAR spatial degrees. The key point behind the IPPM shortage is that, this scheme only requires the initial phase of the transmitted signal linear in azimuth. For the multichannel received echoes, the global coded phases in azimuth are commonly nonlinear. Consequently, it can only carry out Range-Doppler filtering for each receiver individually, but not capable of combining all receiving channels to demodulate.

In this paper, a novel orthogonal waveform separation scheme based on echo-compression is proposed for airborne MIMO-SAR systems. For simplicity, we assume that the transmitted signals are the up and down chirps. Then, for the typical MIMO-SAR signal models, the up and down chirps are supposed to be transmitted simultaneously. However, in the proposed scheme, besides the simultaneously transmissions, the up chirp is required to be radiated several times alone in a synthetic aperture to sense its unambiguous channel responses. Since the channel responses at the neighboring azimuth positions are relevant, the energy of the up chirp for each azimuth time will be mostly concentrated to a peak by matched-filtering the superposed echoes with its closest sensed unambiguous echo. Then, the energy of the up and down chirps can be separated perfectly by cancelling the highest peak from the echo-compression results. Moreover, the cleaned superposed echoes can be used to reconstruct the unambiguous echoes of the down chirp, and the unambiguous echoes of the up chirp can be reconstructed by subtracting the unambiguous down chirp echoes from the original superposed echoes. This proposed scheme is mainly inspired by the cognitive radar concepts. It can ensure a better MIMO-SAR imaging performance with low system complexity and cost. The remainder of this paper is organized as follows: the orthogonality confusion of MIMO-SAR systems is described in Section 2, which is followed by the proposed scheme in Section 3. Simulations are detailed in Section 4.

## 2. Orthogonality Confusion of MIMO-SAR Systems

Since the concept of MIMO-SAR was proposed, an important issue arose with regards to the separation of the scattered radar echoes from the multiple transmitted signals. Several publications have suggested the orthogonal waveforms as solutions. However, what exactly is “orthogonal”?

From the math perspective, the orthogonal condition is

(1) $$\int s_{i}\left(t\right)\cdot s_{j}^{*}\left(t\right)\cdot dt=0,\;\mathrm{if}\;i\neq j$$

where $s_{i}\left(t\right)$ and $s_{j}\left(t\right)$ are the mutually orthogonal signals. 

The mathematical definition mainly requires the inner product of two different signals to be zero. It can separate the multiple waveforms for zero time shifts perfectly, even if they share the same frequency band. To this end, it has been successfully used for the direct wave demodulation in communication systems. However, for radar applications with correlation processing procedure in the receiver, the orthogonal condition with zero time shifts is not sufficient to ensure reliable signal separation. The following orthogonal condition, which demands zero inner product for arbitrary shifts, is more reasonable

(2) $$\int s_{i}\left(t\right)\cdot s_{j}^{*}\left(t-\tau \right)\cdot dt=0,\;\forall \;\tau \in R,\;\mathrm{if}\;i\neq j$$

However, theoretically speaking, as long as $s_{i}\left(t\right)$ and $s_{j}\left(t\right)$ share the same coverage in the time-frequency plane, the condition in Equation (2) will violate the principle of conservation of energy. As a result, some authors have suggested relaxing the constraints as follows

(3) $$\int s_{i}\left(t\right)\cdot s_{j}^{*}\left(t-\tau \right)\cdot dt=\delta ,\;0<\delta <<1,\;\mathrm{if}\;i\neq j$$

The most popular signals fulfilling the relaxed orthogonal condition are the up and down chirps. The amplitude δ in the condition denotes the sidelobes level, namely the spread energy of the mismatched waveforms. Its impact on traditional radar applications that focus on a sparse set of targets is negligible. However, for SAR systems aiming to image every detail in the scene, the ambiguous energy would be accumulated from numerous spatially distributed targets. Consequently, the background noise level will be dramatically increased, and the MIMO-SAR images would be degraded significantly by the orthogonal waveforms fulfilling condition (3).

To express the orthogonality confusion more intuitively, performance of the relaxed orthogonal condition shown in Equation (3) is further analyzed in this section. Assume that the simultaneously transmitted waveforms are up and down chirps, the equivalent baseband echo received by the *n*-th antenna can be expressed as

(4) $$s_{n}\left(t_{r},\eta \right)=\sum_{i=1}^{P}\sum_{m=1}^{2}\sigma_{i}\left[\begin{array}{l} \mathrm{rect}\left(\frac{t_{r}-\tau_{n,m,i}}{T}\right)\cdot \\ \exp\left(j\pi {\left(-1\right)}^{\left(m-1\right)}k_{r}\cdot {\left(t_{r}-\tau_{n,m,i}\right)}^{2}\right)\cdot \\ \exp\left(-j2\pi f_{0}\tau_{n,m,i}\right) \end{array}\right]$$

where the notations are given as follows:


$\tau_{n,m,i}$
Delay of *i*-th scatter that radiated from the *m*-th transmitter and the *n*-th receiver 
$t_{r}$
Fast time
η
Slow time
T
Chirp duration
$k_{r}$
Chirp rate
$f_{0}$
Carrier frequency
P
Number of the closely spaced scatters in the spatially distributed target
$\sigma_{i}$
Radar cross-section (RCS) of the *i*-th scatter

By matched-filtering the echo with the up chirp, we can get the echo that transmitted from the first antenna and received by the *n*-th one

(5) $$s_{n,1}\left(t_{r},\eta \right)=\sum_{i=1}^{P}\sigma_{i}\left[\begin{array}{l} \sqrt{k_{r}T^{2}}\mathrm{sinc}\left(\pi k_{r}T\left(t_{r}-\tau_{n,1,i}\right)\right)\cdot \\ \exp\left(-j2\pi f_{0}\tau_{n,1,i}\right)\\ +\frac{1}{\sqrt{2}}\mathrm{rect}\left(\frac{t_{r}-\tau_{n,2,i}}{2T}\right)\cdot \\ \exp\left(-j\pi \frac{k_{r}}{2}\cdot {\left(t_{r}-\tau_{n,2,i}\right)}^{2}\right)\cdot \\ \exp\left(-j2\pi f_{0}\tau_{n,2,i}\right) \end{array}\right]$$

**Figure 1:**
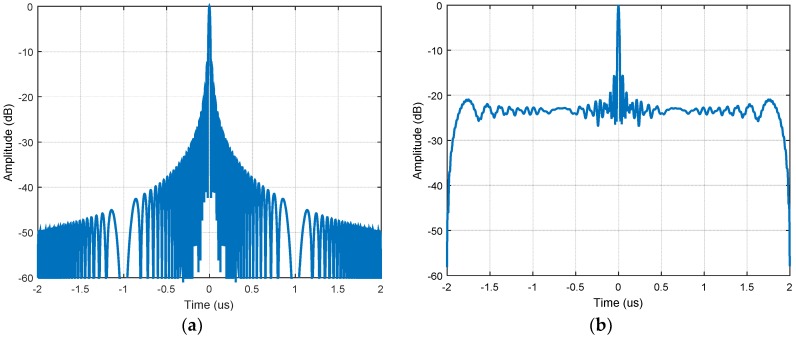
(**a**) Point spread function of the chirp; (**b**) compression result of the superposed echoes.

**Figure 2:**
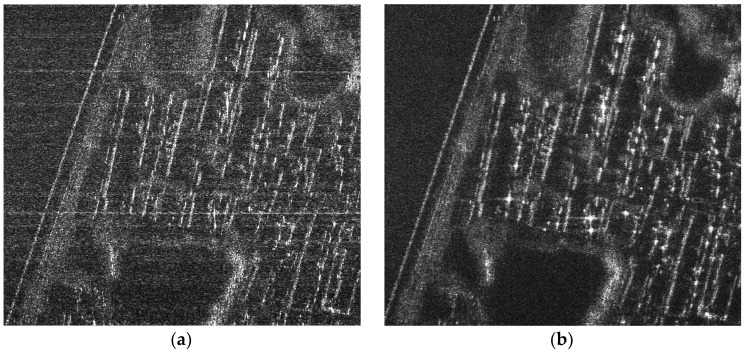
(**a**) MIMO-SAR image with the up and down chirps; (**b**) referenced SAR image.

It can be seen from Equation (5) that, while the up chirp is compressed, the unmatched down chirp has been expanded to 2T. The up and down chirps, which fulfilling the relaxed orthogonal condition, cannot offer perfect waveform separation. The energy of the unmatched down chirp is spread in the range direction (see Figure 1). For the scenario containing spatially distributed targets with numerous closely spaced scatters, the ambiguous energy from each backscatter will be accumulated and increases the background noise level. Consequently, the imperfect waveform separation introduced ambiguous energy degrades SAR image significantly (see Figure 2).

To overcome the fundamental challenges in the orthogonal conditions, multidimensional waveforms such as STSO, OFDM, and IPPM have been proposed. However, as aforementioned, these waveforms would increase the system complexity and PRF substantially. In the following section, we will propose a more practical orthogonal waveform separation scheme with low cost. 

## 3. Orthogonal Waveform Separation Based on Echo-Compression

**Figure 3:**
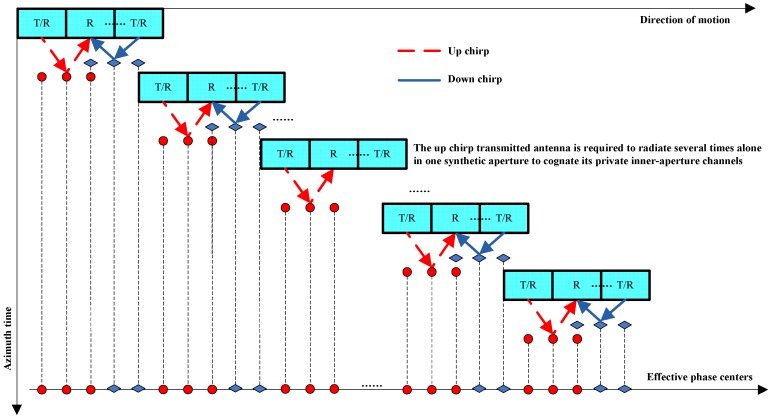
Transmission model of the proposed scheme used for MIMO-SAR system. The up and down chirps are transmitted by the first and the last antennas, respectively. All its antennas are used for receiving echoes. The platform equipped with regularly and compactly spaced antennas is required to move (2N−1)D/2 during one pulse repeat interval (PRI), where *N* denotes the antenna number, and *D* denotes the subantenna length. The up chirp transmitted antenna is required to radiate several times alone in one synthetic aperture to cognate its private inner-aperture channels.

To obtain a perfect separation of the superposed echoes with respect to the multiple transmitted orthogonal waveforms without increasing the system complexity, this paper proposes a novel echo-compression scheme for airborne MIMO-SAR systems based on relevant scene. This scheme is mainly inspired by the cognitive radar concepts [17,18], which continuously learns about the environment through experience gained from interactions with the environment and, in a corresponding way, continually updates the transceivers with relevant information on the environment. For simplicity, we assume that there are two orthogonal waveforms to be transmitted, namely the up and down chirps. Then the proposed scheme consists of three main steps: Firstly, for the system model, apart from the simultaneously transmissions, one of the orthogonal waveforms is required to be radiated several times alone in each synthetic aperture to sense its private inner-aperture channels (see Figure 3). Secondly, for the orthogonal waveform separation, matched-filtering the superposed echoes with the closest sensed echo, and extract the concentrated peaks. Finally, for the unambiguous echo reconstruction, the cleaned and the original superposed echoes, are used to reconstruct both the unambiguous echoes of the multi-transmitted waveforms.

**Figure 4:**
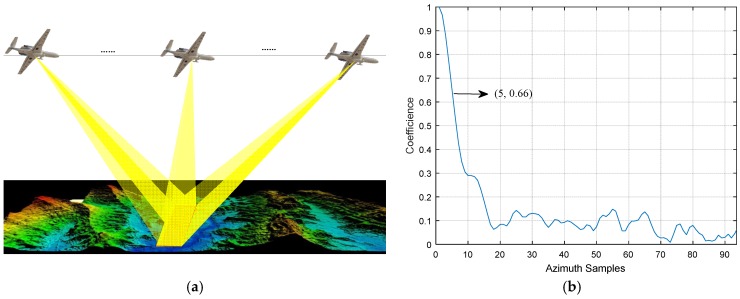
(**a**) MIMO-SAR beam developments for the proposed scheme. Instead of moving in accordance with the platform movement, the radar beams are focusing on the same scene during the whole synthetic aperture; (**b**) channel responses coefficient of a typical airborne SAR scene for the beam development. In this case, if the waveforms are radiated every ten azimuth positions alone, the coefficient can be ensured higher than 0.66, namely, the channel responses of the ten adjacent azimuth positions are relevant.

Please note that, the novel scheme is based on the assumption that the channel responses at the neighboring azimuth positions are relevant. Otherwise, there exists no concentrated peaks in the echo-compression results, and the ambiguous energy cannot be cancelled. To fulfill the relevance assumption, we mainly require the absolute value of the correlation coefficient between the neighboring azimuthal channel responses to be larger than 0.6. The radar beams, as a result, are suggested to focus on the same scene during the whole synthetic aperture (see Figure 4). 

Besides, since the channel response decorrelation introduced by the ionosphere and the atmosphere are significant, the novel scheme is not suitable for spaceborne MIMO-SAR systems. A potential application of the proposed scheme may be the airborne wide-swath spotlight imaging. Due to the increased equivalent phase centers in the azimuth direction (see Figure 3), the system PRF can be reduced by a factor of 2N−1 to cover a wider swath with lower range ambiguities. This PRF reduction would not cause azimuth ambiguities either, because the equivalent PRF are required to be larger than the Doppler bandwidth. If compared to the time-division multiplexing (TDM)-based MIMO systems with two transmitters and two time slots, which require the system PRF to be twice of the Doppler bandwidth, the performance of the proposed scheme for wide-swath spotlight imaging would be increased by a factor of 2(2N−1). Another application may be the airborne 3D imaging. By arranging the MIMO antennas in the elevation direction, we can double the 3D imaging baseline, and get N−1 more equivalent phase centers than the conventional single-input multi-output (SIMO) SAR systems. Then, the resolution in the third dimension of the 3D SAR images can be increased by a factor of two. In this paper, we mainly focus on the MIMO signal model of the proposed scheme. The applications, which may involve wide-swath spotlight imaging and 3D imaging, will be further investigated in the future work.

Assume that the mutually orthogonal waveform are $x_{1}\left(t_{r}\right)$ and $x_{2}\left(t_{r}\right)$, where $x_{1}\left(t_{r}\right)$ denotes the up chirp and $x_{2}\left(t_{r}\right)$ the down chirp. The scene RCS referring to the azimuth time η and the different transmitters are $\sigma_{1}\left(t_{r},\eta \right)$ and $\sigma_{2}\left(t_{r},\eta \right)$. Then, for a given azimuth time $\eta_{1}$ nearing the sensed one $\eta_{2}$, the echo received by the *n*-th antenna is

(6) $$s_{n}\left(t_{r},\eta_{1}\right)=\sigma_{1}\left(t_{r},\eta_{1}\right){⊗}_{t_{r}}x_{1}\left(t_{r}\right)+\sigma_{2}\left(t_{r},\eta_{1}\right){⊗}_{t_{r}}x_{2}\left(t_{r}\right)$$

where ${⊗}_{t_{r}}$ denotes convolution for $t_{r}$.

Transform (6) into the range frequency domain, we can get

(7) $$S_{n}\left(f_{r},\eta_{1}\right)=\sigma_{1}\left(f_{r},\eta_{1}\right)\cdot X_{1}\left(f_{r}\right)+\sigma_{2}\left(f_{r},\eta_{1}\right)\cdot X_{2}\left(f_{r}\right)$$

Based on the above signal model, the unambiguous echo related to $x_{2}\left(t_{r}\right)$ can be separated from the superposed echoes $s_{n}\left(t_{r},\eta_{1}\right)$ by the following three steps:

(1) Matched-filter the superposed echo $s_{n}\left(t_{r},\eta_{1}\right)$ with the sensed echo $\sigma_{1}\left(t_{r},\eta_{2}\right){⊗}_{t_{r}}x_{1}\left(t_{r}\right)$. The echo-compression results are
  (8) $$\begin{array}{ll} S_{n}\left(f_{r},\eta_{1}\right) & =\left[\sigma_{1}\left(f_{r},\eta_{1}\right)\cdot X_{1}\left(f_{r}\right)+\sigma_{2}\left(f_{r},\eta_{1}\right)\cdot X_{2}\left(f_{r}\right)\right]\cdot \sigma_{1}^{*}\left(f_{r},\eta_{2}\right)\cdot X_{1}^{*}\left(f_{r}\right)\\ & ={\left|\sigma_{1}\left(f_{r},\eta_{1}\right)\right|}^{2}+\Delta +\sigma_{2}\left(f_{r},\eta_{1}\right)\cdot X_{2}\left(f_{r}\right)\sigma_{1}^{*}\left(f_{r},\eta_{2}\right)\cdot X_{1}^{*}\left(f_{r}\right) \end{array}$$
  for $\Delta =\sigma_{1}^{*}\left(t_{r},\eta_{1}\right)-\sigma_{1}^{*}\left(t_{r},\eta_{2}\right)$, where ${\left|\sigma_{1}\left(f_{r},\eta_{1}\right)\right|}^{2}$ denotes the ambiguous energy, and ${\left|\Delta \right|}^{2}$ is the residual ambiguous energy. Since the channel responses at the neighboring azimuth positions are supposed to be relevant, the residual ambiguous energy ${\left|\Delta \right|}^{2}$ is extremely low. Then, we can neglect the residual ambiguous energy, and rewrite (8) as follows
  (9) $$S_{n}\left(f_{r},\eta_{1}\right)\approx {\left|\sigma_{1}\left(f_{r},\eta_{1}\right)\right|}^{2}+\sigma_{2}\left(f_{r},\eta_{1}\right)\cdot X_{2}\left(f_{r}\right)\sigma_{1}^{*}\left(f_{r},\eta_{2}\right)\cdot X_{1}^{*}\left(f_{r}\right)$$
(2) Transform (9) into the range time domain and cancel the highest peak to eliminate the ambiguous energy ${\left|\sigma_{1}\left(f_{r},\eta_{1}\right)\right|}^{2}$ from the superposed echoes. The peak cancelled data in the range frequency domain is
  (10) $${S^{\prime }}_{n}\left(f_{r},\eta_{1}\right)=\sigma_{2}\left(f_{r},\eta_{1}\right)\cdot X_{2}\left(f_{r}\right)\sigma_{1}^{*}\left(f_{r},\eta_{2}\right)\cdot X_{1}^{*}\left(f_{r}\right)$$
(3) Multiply (10) with $1/\left[\sigma_{1}^{*}\left(f_{r},\eta_{2}\right)\cdot X_{1}^{*}\left(f_{r}\right)\right]$, and transform the results in to the range time domain. Then, we can get the unambiguous echo related to $x_{2}\left(t_{r}\right)$ as follows
  (11) $$s_{n,2}\left(t_{r},\eta_{1}\right)=\sigma_{2}\left(t_{r},\eta_{1}\right){⊗}_{t_{r}}x_{2}\left(t_{r}\right)$$

As for the unambiguous echo related to $x_{1}\left(t_{r}\right)$, it can be reconstructed by subtracting $s_{n,2}\left(t_{r},\eta_{1}\right)$ from the original superposed echoes $s_{n}\left(t_{r},\eta_{1}\right)$, namely

(12) $$s_{n,1}\left(t_{r},\eta_{1}\right)=s_{n}\left(t_{r},\eta_{1}\right)-s_{n,2}\left(t_{r},\eta_{1}\right)=\sigma_{1}\left(t_{r},\eta_{1}\right){⊗}_{t_{r}}x_{1}\left(t_{r}\right)$$

**Figure 5:**
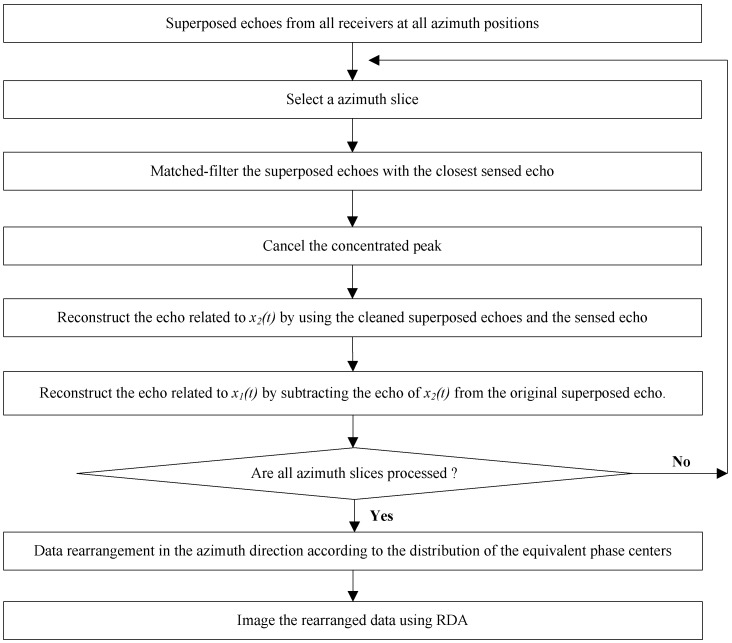
Procedure of the proposed scheme used for MIMO-SAR orthogonal waveform separation.

It can be seen from Equations (11) and (12) that, for a given azimuth time $\eta_{1}$ nearing the sensing one $\eta_{2}$, the superposed echoes received by the *n*-th antenna have been separated. Furthermore, the above algorithm should be performed for each receiver and each azimuth time to obtain all the unambiguous echoes. To this end, we can rearrange the unambiguous echoes in the azimuth direction according to the distribution of the equivalent phase centers, and image the rearranged data with the classical Range-Doppler algorithm (RDA) to get the MIMO-SAR images without ambiguous energy. The procedure of the proposed scheme is detailed in Figure 5. 

It can be seen from Figure 5 that, compared to the multidimensional waveforms such as the OFDM chirp and the STSO waveforms, which are the most promising waveforms for MIMO-SAR systems, the proposed scheme does not need to be combined with the DBF techniques in the elevation direction. To this end, the large amounts of subarrays for the spatial filtering in the systems employing the multidimensional waveforms can be replaced by only one antenna in the elevation. We can ensure a low system complexity and cost without degrading the MIMO-SAR performances. Lastly, it should be noticed that, the proposed scheme is based on but not limited to the up and down chirps. It has no restrictions on the orthogonal waveforms. As long as the channel responses at the neighboring azimuth positions are relevant, any radar waveforms set that fulfilling the relaxed orthogonal condition in Equation (3) can be employed. Actually, if the MIMO system has three or more transmitters, the transmission model and the signal processing procedure are similar to that shown in Figure 3 and Figure 5. In particular, all the waveforms are required to be radiated alone several times in a synthetic aperture to sense their private inner-aperture channels. Then, for a certain waveform, the ambiguous energy can be suppressed by matched-filtering the superposed echo with its closest sensed echoes of all the other waveforms and cancelling the concentrated peaks successively. Through these approaches, the unambiguous echoes of all the waveforms at all the azimuth positions can be reconstructed. In addition, we can get the images without ambiguous energy.

## 4. Simulation Results

**Figure 6:**
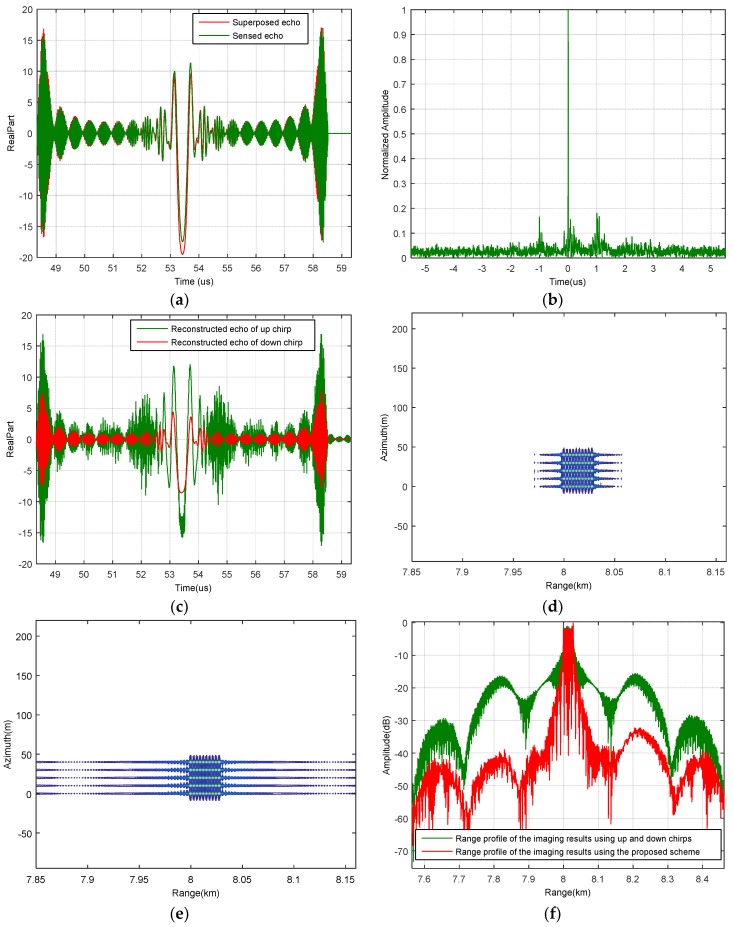
(**a**) The superposed echo received at the 41th azimuth position and the private echo sensed at the 60th azimuth position; (**b**) compression result of the superposed and the sensed echoes; (**c**) reconstructed echoes of the up and the down chirps at the 41th azimuth position; (**d**) imaging result using all the reconstructed signals; (**e**) imaging result using the conventional up and down chirps; (**f**) range profiles of the imaging results shown in (**d**,**e**).

To validate the proposed scheme, simulation with two transceivers in the azimuth direction is designed. Parameters are listed in Table 1. It is assumed that there are 50 scatters in the scene. The distances between the scatters in the range direction and the azimuth direction are 3 m and 10 m, respectively. All the amplitudes of the scatters have been normalized to one. According to the simulation parameters, there are 362 azimuth positions in total. The up chirp is radiated alone at the 20th, 60th, 100th, 140th, 180th, 220th, 260th and 300th azimuth position to sense its private channel responses. Then, for each azimuth position, the superposed echoes are matched-filtered by their closest sensed echo. For instance, the superposed echo received at the 41th azimuth time and the private echo sensed at the 60th azimuth time are shown in Figure 6a. Their compression result is shown in Figure 6b. Since the channel responses are relevant, the energy of the superposed echo has been mainly concentrated. If we cancel the highest peak, the energy of the down chirp can be mostly removed, and the unambiguous echoes of the up and down chirps can be reconstructed (see Figure 6c). Imaging result of the proposed scheme is shown in Figure 6d. Compared with that of the conventional up and down chirps shown in Figure 6e, the background noise level is reduced dramatically. This effect is more apparent in the range profiles shown in Figure 6f. Please note that, since the up chirp is required to radiate alone for several times, the overall equivalent phase centers will be reduced. However, the reduced phase centers can be ignored because of the massive phase centers in the azimuth direction. 

**Figure 7:**
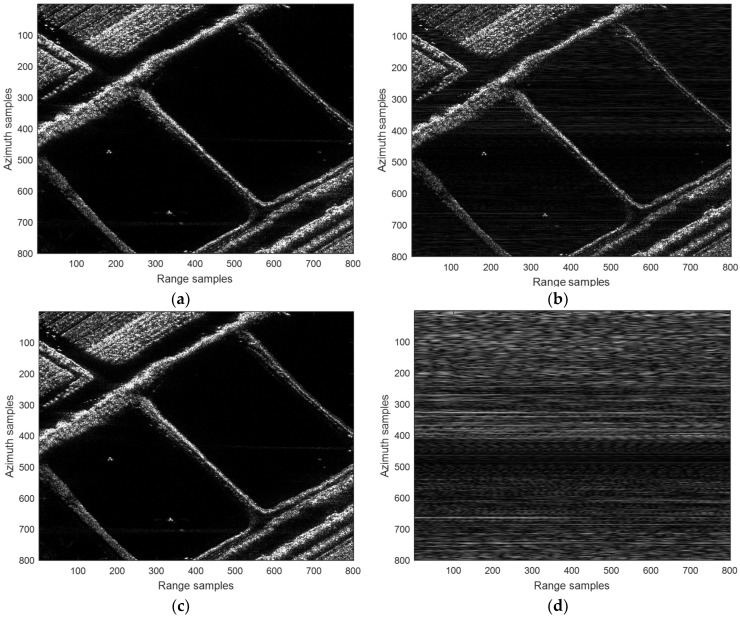

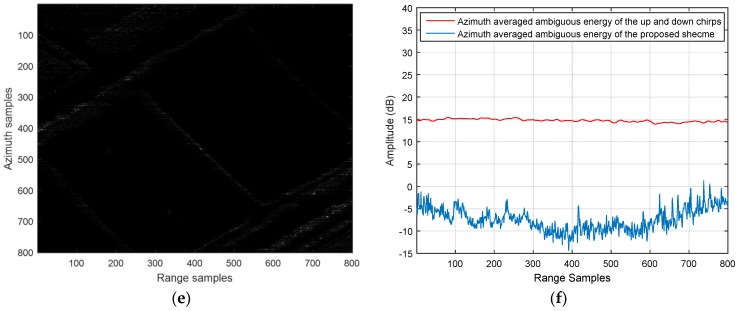
(**a**) Original scene; (**b**) MIMO-SAR image using the conventional up and down chirps; (**c**) MIMO-SAR image using the proposed echo-compression scheme; (**d**) the ambiguous energy in (**b**); (**e**) the ambiguous energy in (**c**); (**f**) the azimuth averaged ambiguous energy in (**d**,**e**).

To further validate the proposed scheme, a real-scene simulation based on a C-band MIMO-SAR system is designed. The echoes in the simulation are reconstructed according to a practical airborne SAR dataset. Parameters used in the simulation are the same as those listed in Table 1. Simulation results are shown in Figure 7. In particular, Figure 7a is the original scene. It can be seen from Figure 7b that, the up and down chirps, which fulfill the relaxed orthogonal condition, is not suitable for MIMO-SAR imaging. The energy of the mismatched chirp is spread but not removed in the range direction. It has been accumulated from numerous closely spaced scatters. As a result, the background noise level is increased, and the MIMO-SAR images are seriously degraded. However, by employing the proposed echo-compression scheme, the echoes of the imperfect orthogonal waveforms can be separated to a large extent. Then, the ambiguous energy is reduced, and in Figure 7c, we can get a better background noise level. Based on the real-scene simulation results, quantitative comparisons between the performance of the proposed scheme and that of the conventional up and down chirps are performed. In particular, the images of the ambiguous energy are firstly formed by subtracting the reconstructed MIMO-SAR images from the original scene, respectively. Then, the root mean square error (RMSE) are computed according to the subtraction results. It can be seen from Figure 7d,e that the ambiguous energy of the up and down chirps is dramatically high than that of the proposed scheme. Consequently, the RMSE are 5.45 and 0.47, respectively. The performance comparison is more intuitive in the image of the azimuth averaged ambiguous energy (see Figure 7f). 

Through the above point-targets and real-scene simulations, we can conclude from that the novel orthogonal waveform separation scheme for airborne MIMO-SAR systems has been validated.

## 5. Conclusions and Future Directions

In this paper, we proposed a novel orthogonal waveform separation scheme for airborne MIMO-SAR systems. This scheme, which is inspired by the cognitive radar concepts, can provide perfect waveform separation with low system complexity and cost, and enhance MIMO-SAR performance by reducing the pulse repeat frequency. Theoretical analysis has been validated by simulation results. Our future work includes exploiting the advantages of the scheme by using a practical airborne MIMO-SAR system in wide-swath spotlight and 3D imaging [19,20]. 

## Figures and Tables

**Table 1:** Parameters used in the simulations.

Antenna number	2
Antenna length	3 m
Pulse duration	10 µs
Bandwidth	100 MHz
Carrier frequency	5.4 GHz
Sampling frequency	120 MHz
Platform velocity	150 m/s
Slant range	8 km
Doppler bandwidth	100 Hz
System PRF	50 Hz
Equivalent PRF	150 Hz
